# Human Cardiac-Derived Adherent Proliferating Cells Reduce Murine Acute Coxsackievirus B3-Induced Myocarditis

**DOI:** 10.1371/journal.pone.0028513

**Published:** 2011-12-09

**Authors:** Kapka Miteva, Marion Haag, Jun Peng, Kostas Savvatis, Peter Moritz Becher, Martina Seifert, Katrin Warstat, Dirk Westermann, Jochen Ringe, Michael Sittinger, Heinz-Peter Schultheiss, Carsten Tschöpe, Sophie Van Linthout

**Affiliations:** 1 Berlin-Brandenburg Center for Regenerative Therapies, Charité, University Medicine Berlin, Campus Virchow, Berlin, Germany; 2 Laboratory for Tissue Engineering, Charité, University Medicine Berlin, Berlin, Germany; 3 Department of Cardiology and Pneumology, Charité, University Medicine Berlin, Campus Benjamin Franklin, Berlin, Germany; 4 Institute of Medical Immunology, Charité, University Medicine Berlin, Germany; Heart Center Munich, Germany

## Abstract

**Background:**

Under conventional heart failure therapy, inflammatory cardiomyopathy typically has a progressive course, indicating a need for alternative therapeutic strategies to improve long-term outcomes. We recently isolated and identified novel cardiac-derived cells from human cardiac biopsies: cardiac-derived adherent proliferating cells (CAPs). They have similarities with mesenchymal stromal cells, which are known for their anti-apoptotic and immunomodulatory properties. We explored whether CAPs application could be a novel strategy to improve acute Coxsackievirus B3 (CVB3)-induced myocarditis.

**Methodology/Principal Findings:**

To evaluate the safety of our approach, we first analyzed the expression of the coxsackie- and adenovirus receptor (CAR) and the co-receptor CD55 on CAPs, which are both required for effective CVB3 infectivity. We could demonstrate that CAPs only minimally express both receptors, which translates to minimal CVB3 copy numbers, and without viral particle release after CVB3 infection. Co-culture of CAPs with CVB3-infected HL-1 cardiomyocytes resulted in a reduction of CVB3-induced HL-1 apoptosis and viral progeny release. In addition, CAPs reduced CD4 and CD8 T cell proliferation. All CAPs-mediated protective effects were nitric oxide- and interleukin-10-dependent and required interferon-γ. In an acute murine model of CVB3-induced myocarditis, application of CAPs led to a decrease of cardiac apoptosis, cardiac CVB3 viral load and improved left ventricular contractility parameters. This was associated with a decline in cardiac mononuclear cell activity, an increase in T regulatory cells and T cell apoptosis, and an increase in left ventricular *interleukin-10* and *interferon-γ* mRNA expression.

**Conclusions:**

We conclude that CAPs are a unique type of cardiac-derived cells and promising tools to improve acute CVB3-induced myocarditis.

## Introduction

Myocarditis is a common inflammatory cardiomyopathy, associated with cardiomyocyte apoptosis, which can lead to chronic left ventricular (LV) dysfunction. Infection of mice with Coxsackievirus B3 (CVB3) is the most common experimental model of myocarditis and has provided important insights into the pathogenesis of human disease. CVB3 causes cardiomyocyte apoptosis via its direct cytopathic effects [1], [2] as well as via immune-mediated mechanisms [3], [4]. Under conventional heart failure therapy, inflammatory cardiomyopathy typically has a progressive course, indicating a need for alternative therapeutic strategies to improve long-term outcomes.

Experimental [5], [6] and clinical studies [7], [8] have consistently supported the application of cellular transplantation as a strategy to improve myocardial function [6], [9]. Whereas experimental studies [10] as well as clinical trials [9] have been performed with stem cells for the treatment of myocardial infarction or chronic myocardial ischemia, only few experimental cell-based studies are directed at treating nonischemic cardiomyopathies [6], [11]. We recently isolated and identified novel cardiac-derived cells from human cardiac biopsies: cardiac-derived adherent proliferating cells (CAPs) characterized as CD105^+^, CD73^+^, CD166^+^, CD44^+^, CD90^−^, CD14^−^, CD34^−^ and CD45^−^ [12], [13]. CAPs have similarities with mesenchymal stromal cells (MSCs), which are known for their anti-apoptotic [11] and immunomodulatory [14] features and have been shown to reduce CVB3-induced [15] and autoimmune [16] myocarditis. MSCs suppress T cell responses [17], [18], induce apoptosis of activated T cells [19] and increase T regulatory cells [20]. As in the case of MSCs, CAPs are low immunogenic [21], whereas in contrast to MSCs, CAPs do not have a multilineage differentiation potential.

The present study explores whether CAPs share these anti-apoptotic and immunomodulatory features with MSCs and whether they are potential agents for the treatment of acute CVB3-induced inflammatory cardiomyopathy. To address potential safety concerns, we first investigated whether CAPs express the Coxsackie- and adenovirus receptor (CAR) [22] and the co-receptor CD55 [23], which are both necessary for effective CVB3 infectivity. Furthermore, we analyzed whether and how CAPs can reduce CVB3-induced HL-1 cardiomyocyte apoptosis, viral progeny release, and T cell activation *in vitro* and whether our findings can be extrapolated into a murine experimental model of acute CVB3-induced myocarditis.

## Results

### Cardiac-adherent proliferating cells minimally express the Coxsackie- and adenovirus receptor and co-receptor CD55

**Figure 1:**
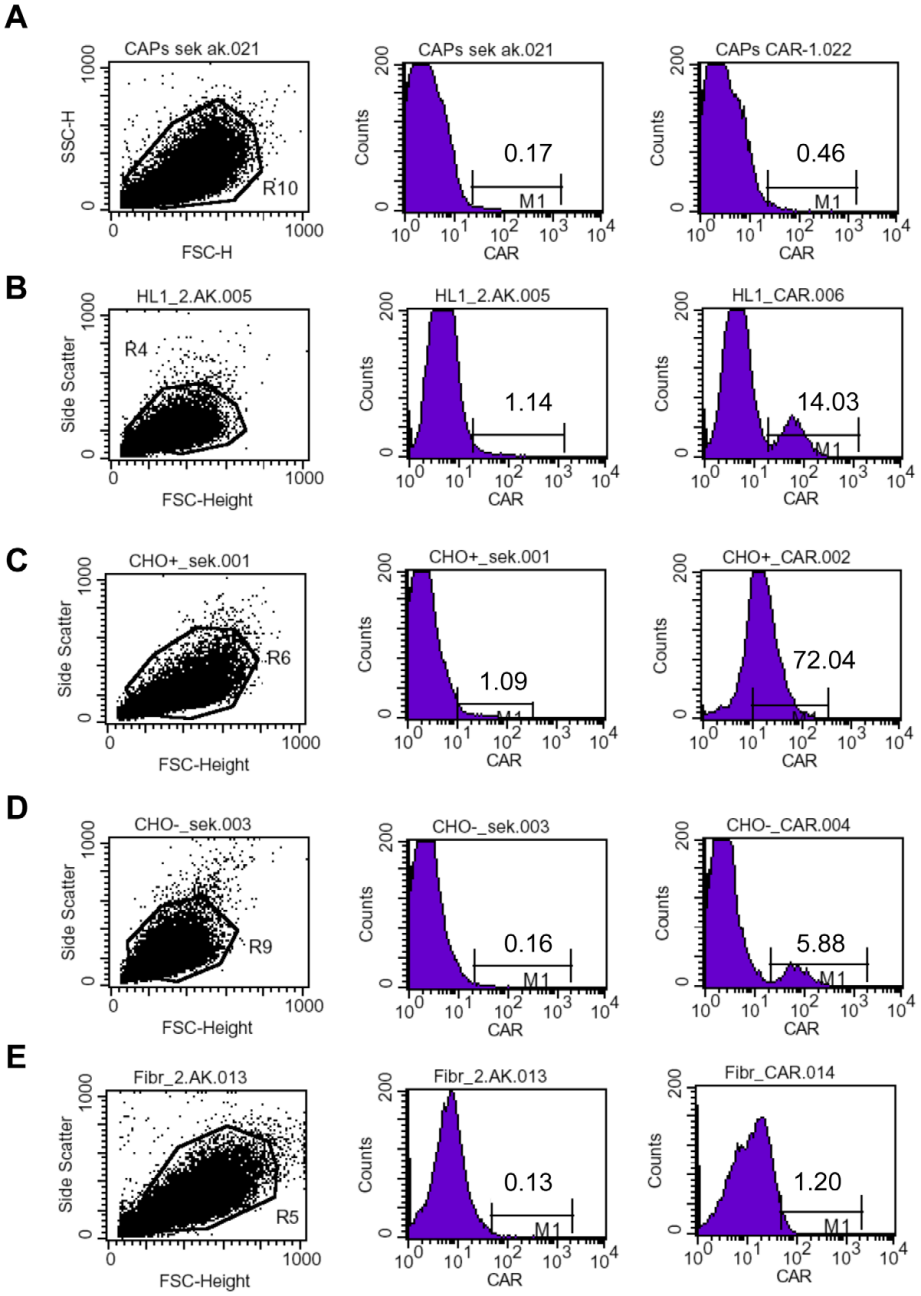
Cardiac adherent proliferating cells minimally express Coxsackievirus-adenovirus receptor. Representative pictures of the Forwards scatter (FSC)/Sideward scatter (SSC) (left), of the percentage of secondary antibody positive cells (middle), and of the percentage of CAR positive cells (right) are shown for **A.** CAP cells, **B.** HL-1 cells, **C.** CHO-CAR cells, **D.** CHO cells, and **E.** cardiac fibroblasts.

**Figure 2:**
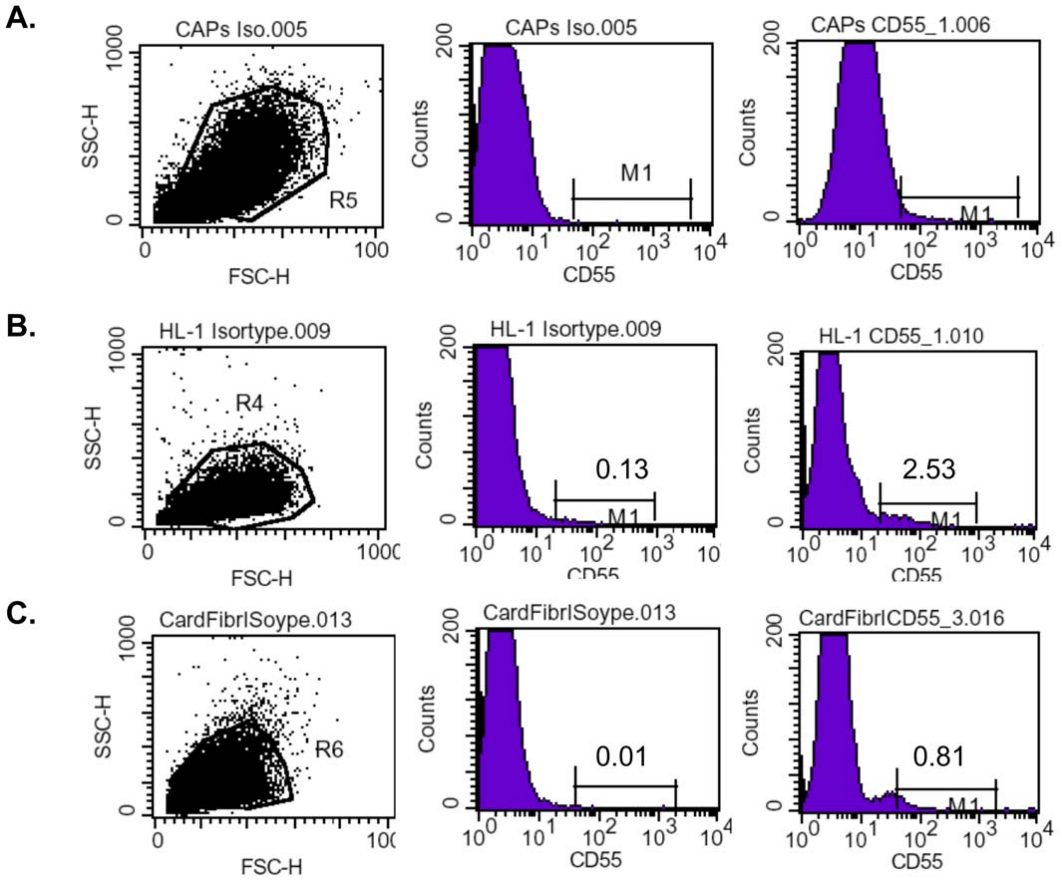
Cardiac adherent proliferating cells minimally express CD55. Representative pictures of the Forwards scatter (FSC)/Sideward scatter (SSC) (left), of the percentage of isotype control positive cells (middle), and of the percentage of CD55 positive cells (right) are shown for **A.** CAP cells, **B.** HL-1 cells, and **C.** cardiac fibroblasts.

Cardiac adherent proliferating cells (CAPs) were isolated from endomyocardial biopsies [12] taken from the right ventricle side of the interventricular septum [24] of 3 patients after their written approval. A representative surface expression profile of a multicolor flow cytometry analysis of CAPs is shown in **Figure S1**. Given the importance of CAR [22] and CD55 [23] for the infectivity of cells by CVB3, our first point of interest was to investigate whether CAPs express CAR and CD55. As positive controls for CAR expression, we used Chinese Hamster Ovary (CHO) cells, which were stably transfected with CAR and which overexpress CAR (CHO-CAR) [25], and murine HL-1 cells, since cardiomyocytes are the target cells of CVB3. As negative controls, we used CHO cells, which lacked the CAR receptor, and cardiac fibroblasts, since it has been reported that primary fibroblasts only express low levels of CAR [26]. Compared to CHO-CAR and HL-1 cells, as well as to CHO and cardiac fibroblasts, CAPs only minimally express CAR at levels nearly comparable with secondary antibody controls (**Figure 1**). As for CAR, CAPs also only moderately express CD55 (**Figure 2**).

### Effect of Coxsackievirus B3 infection on cardiac adherent proliferating cell viability

**Figure 3:**
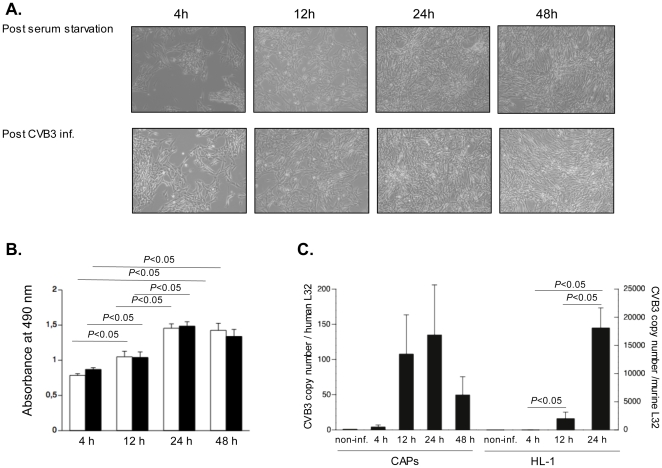
Cell viability of cardiac adherent proliferating cells is not hampered by Coxsackievirus B3-infection. CAPs were plated in a 6-well for phase contrast pictures and CVB3 copy number analysis or in a 96-well for MTS assay, respectively. After 24 h, reaching 80% confluence, CAPs were infected with CVB3 at an m.o.i. of 5 or serum starved. Four, 12, 24 and 48 h after infection or serum starvation, phase contrast pictures were taken or an MTS assay was performed. CAPs were collected 4, 12, 24 and 48 h after infection for CVB3 RNA analysis. **A.** Phase contrast pictures of CAPs, 4 h, 12 h, 24 h and 48 h after serum starvation (upper panel) or CVB3 infection (lower panel), at 100× magnification. **B.** Bar graphs representing the absorbance at 490 nm from uninfected (open bar graphs) and CVB3-infected (closed bar graphs) CAPs 4 h, 12 h, 24 h and 48 h after serum starvation or CVB3-infection, respectively. n = 6/group. **C.** Bar graphs representing CVB3 copy number in CVB3-infected CAPs 4 h, 12 h, 24 h, and 48 h after CVB3-infection expressed as CVB3 copy number versus human L32 (n = 3/condition) and in CVB3-infected HL-1 4 h, 12 h, and 24 h after CVB3-infection (n = 4/condition) expressed as CVB3 copy number versus human L32.

Phase contrast pictures did not show any significant changes in cell morphology between CVB3-infected CAPs and serum-starved controls (**Figure 3A**). In line with this observation, no significant differences were found in CAPs viability between post-infection versus post-serum starvation treatments at all time-points (**Figure 3B**). In agreement with the low CAR and CD55 expression, which suggests low CVB3 uptake, CVB3 copy number presence in CAPs was minimal (**Figure 3C**). In contrast to HL-1 cells in which the CVB3 RNA copy number increased over time, indicating CVB3 replication, the CVB3 copy number tendentially declined in CAPs following CVB3-infection (**Figure 3C**). Importantly, no plaques were detected on HeLa cells incubated with the medium collected from CAPs 24 h after CVB3 infection, indicating the absence of viral progeny release.

### Cardiac-adherent proliferating cells reduce Coxsackievirus B3-induced apoptosis

**Figure 4:**
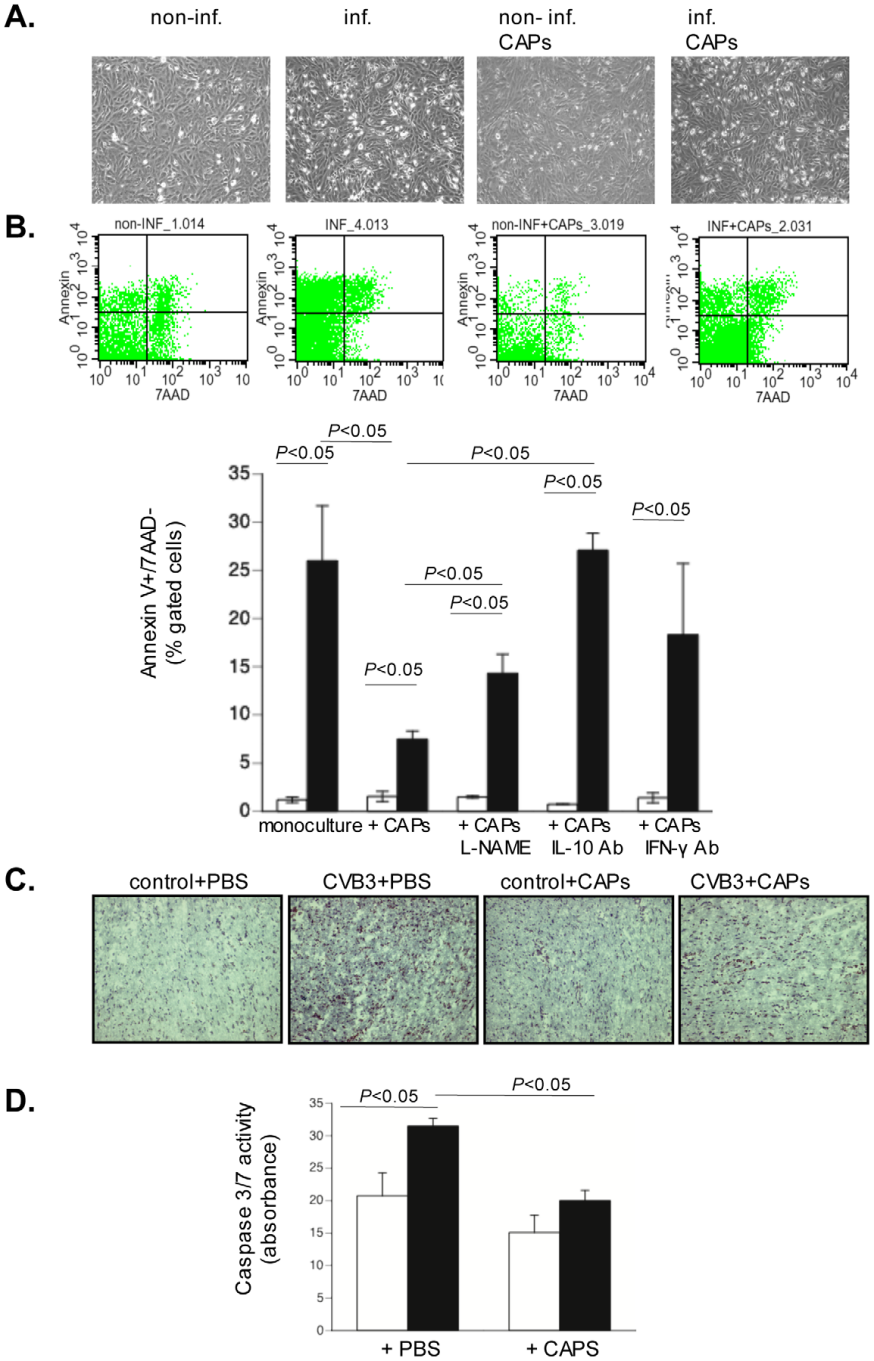
Cardiac adherent proliferating cells reduce Coxsackievirus B3-induced HL-1 apoptosis in a nitric oxide- and interleukin-10-dependent manner and require interferon-γ priming. DiO-labeled HL-1 cells were cultured in a 6-well plate and infected 24 h later with CVB3 at an m.o.i. of 5 for 1 h. Four hours afterwards, CAPs were added at a ratio of 1 to 10 HL-1 cells. After 24 hours, cells were collected for Annexin V/7AAD FACS analysis. **A.** Panel shows representative pictures of uninfected HL-1, infected HL-1, uninfected HL-1 co-cultured with CAPs and infected HL-1 co-cultured with CAPs. **B.** Panel demonstrates representative pictures of Annexin V/7AAD dot plots on preselected DiO+ HL-1 cells. Bar graphs represent DiO+ Annexin V+/7AAD- HL-1 cells in cultures of uninfected (open bars) or CVB3-infected (closed bars) HL-1 with or without untreated or L-NAME treated CAPs or CAPs in the presence or absence of 1 µg/ml of anti-human IL-10 antibody (ab), or 1 µg/ml of anti-murine IFN–γ ab; n = 4/group. **C.** Representative pictures of TUNEL-stained heart sections (from left to right) of control and CVB3-infected mice receiving PBS (control+PBS and CVB3+PBS, respectively) and of control or CVB3-infected mice receiving CAPs (control+CAPs and CVB3+CAPs, respectively), at 200× magnification. **D.** Bar graphs represent the mean ± SEM of caspase 3/7 activity in LV homogenates of control mice (open bars) and CVB3-infected mice (closed bars) injected with PBS or CAPs; n = 5–7/group.

To investigate whether CAPs can reduce CVB3-induced cardiomyocyte apoptosis, we co-cultured CAPs with CVB3-infected Dil-labeled HL-1 and performed Annexin V/7AAD flow cytometry analysis. Phase contrast pictures showed that CAPs reduce the amount of floating cells due to CVB3 induction, indicating a decrease in necrotic cells (**Figure 4A**). In addition, CAPs decreased CVB3-induced HL-1 apoptosis by 3.5-fold (p<0.05) (**Figure 4B**). Since NO exerts anti-apoptotic effects on cardiomyocytes [27], [28] and has anti-viral properties [29], and since IL-10 has been reported to reduce cardiomyocyte apoptosis [30], we further investigated whether CAPs perform their anti-apoptotic effects in an NO- and/or IL-10 dependent way by pre-treating CAPs with the NO-inhibitor L-NAME or co-culturing the HL-1 and CAPs in the presence of an anti-human IL-10 antibody. We showed that under both conditions, the CAPs-mediated anti-apoptotic effects were less pronounced. Subsequently, we analyzed whether CAPs require IFN-γ to exert their protective effects, by co-culturing CAPs with HL-1 in the presence of an anti-mouse IFN-γ-neutralizing antibody and again found a reduction in anti-apoptotic CAPs activity (**Figure 4B**). To investigate the role of IL-10 release in the CAPs-mediated anti-apoptotic effects, 6 pg/ml of recombinant human IL-10, corresponding to the concentration of human IL-10 released by CAPs in HL-1/CAPs co-cultures, was used to treat HL-1 cells. IL-10 decreased the percentage of CVB3-induced Annexin V+/7AAD- HL-1 cells by 1.3-fold (p<0.05) (Annexin V+/7AAD- (% gated cells) in control: 3.7±0.33, control+IL-10: 2.8±0.41, CVB3: 29±2.7, CVB3+IL-10: 23±0.88). In line with our *in vitro* findings, application of CAPs in an experimental model of murine acute myocarditis reduced cardiac apoptosis as shown by TUNEL staining (**Figure 4C**) and as indicated by a 1.6-fold (p<0.01) decrease in caspase 3/7 activity to levels not significantly different from controls (**Figure 4D**).

### Cardiac-adherent proliferating cells reduce viral progeny release

**Figure 5:**
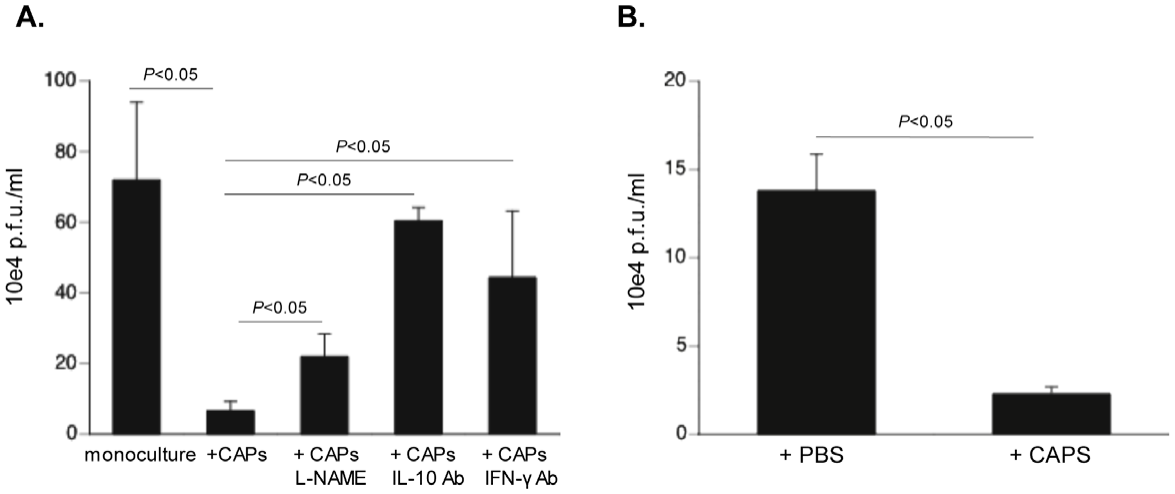
Cardiac adherent proliferating cells reduce Coxsackievirus B3 viral progeny release in a nitric oxide- and interleukin-10-dependent manner and require interferon-γ priming. HeLa cells were incubated for 30 minutes with 1 ml of diluted supernatant of CVB3-infected HL-1 cells or HL-1 cells co-cultured with CAPs, in the presence or absence of L-NAME, anti-human IL-10 antibody, or anti-murine IFN-γ antibody, or with diluted LV homogenates from CVB3 or CVB3+CAPs mice. Then, cells were washed with PBS and covered with agar consisting of 50% 1.3% noble agar and 50% 2× MEM, supplemented with 4% FBS. **A.** Bar graphs represent the mean ± SEM of plaques counted in 6 wells/condition, 72 h after incubation with diluted medium (all at dilution 10^−4^) of CVB3-infected HL-1 cells or HL-1 cells co-cultured with CAPs, in the presence or absence of L-NAME, 1 µg/ml of anti-human IL-10 antibody (Ab), or 1 µg/ml of anti-murine IFN-γ Ab. **B.** Bar graphs represent the mean ± SEM of plaques counted in 12 wells/group (3 wells/mouse and n = 4 mice/group), 72 h after incubation with diluted medium (all at dilution 10^−4^) of CVB3+PBS or CVB3+CAPs mice as indicated.

To analyze whether CAPs could directly affect viral load in CVB3-infected HL-1 cells and to investigate the underlying mechanisms, CAPs were co-cultured with CVB3-infected HL-1 cells and the effect of CAPs on CVB3 viral progeny release was analyzed. Plaque assay demonstrated that CAPs reduced the viral CVB3 titer by 11–fold (p<0.05), an effect, which was blunted in the presence of L-NAME or by blocking either human IL-10 or murine IFN-γ (**Figure 5A**). *In vivo*, CAP administration reduced cardiac viral load by 5.2-fold (p<0.05) (**Figure 5B**) as determined by plaque assay on extracts of LV from CVB3+PBS mice and CVB3+CAPs-injected mice.

### Cardiac-adherent proliferating cells exert immunomodulatory effects

**Figure 6:**
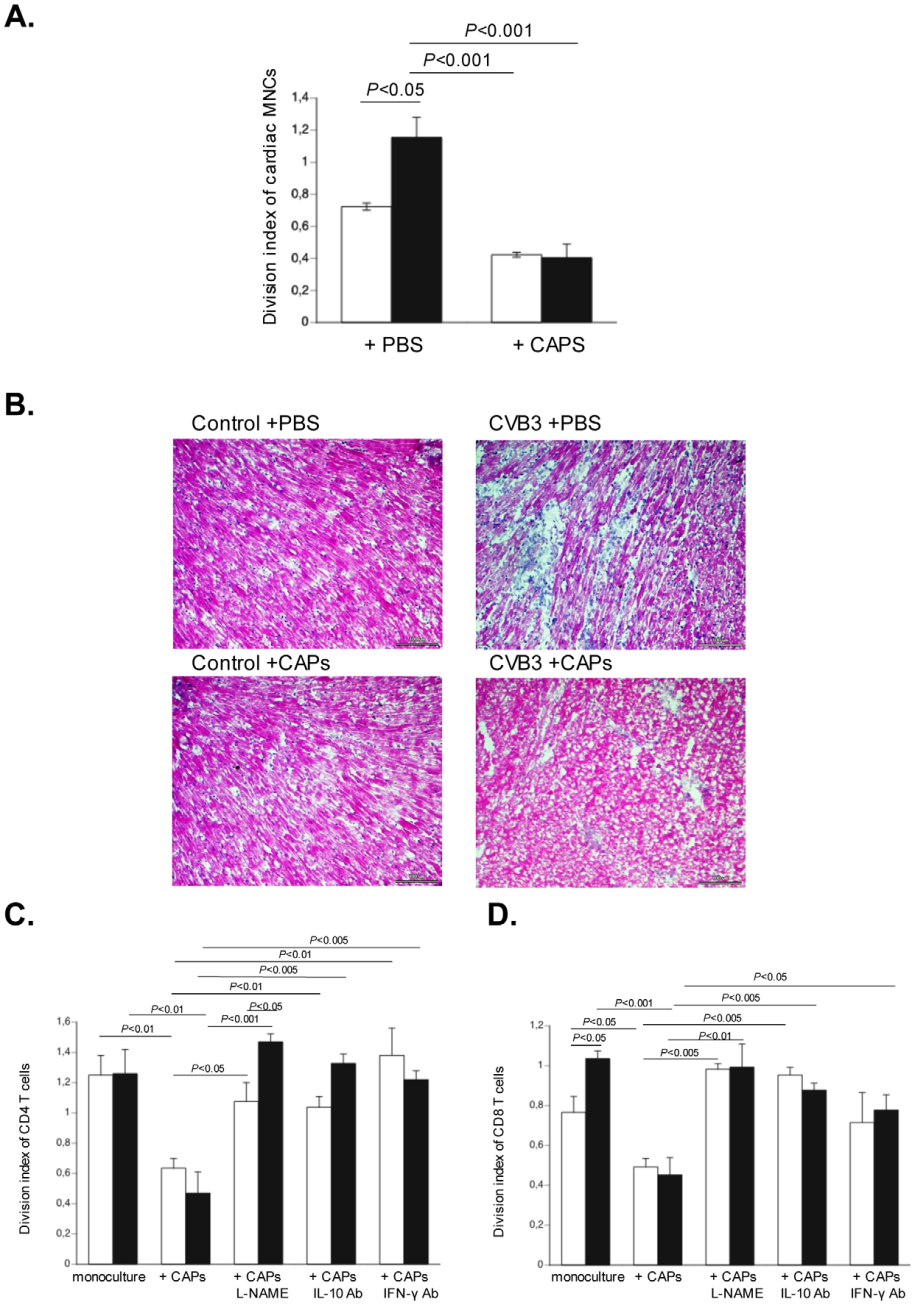
Cardiac adherent proliferating cells decrease cardiac mononuclear cell activation and cardiac damage in Coxsackievirus B3-infected mice. **A.** Cardiac MNCs were isolated from control mice (open bars) and CVB3-infected mice (closed bars) injected with PBS or CAPs. Next, cardiac MNCs were labeled with 10 µM of carboxyfluorescein succinimidyl ester to be able to measure cell proliferation, and stimulated with phorbol myristate acetate (PMA) and ionomycin at a final concentration of 50 ng/ml and 500 ng/ml, respectively, for 72 h followed by flow cytometry and analysis with FlowJo 8.7. software. Bar graphs represent the division index of cardiac MNCs with n = 4/group. **B.** Representative hematoxylin and eosin stained heart sections of control mice receiving PBS (control+PBS; upper left panel) or CAPs (control+CAPs; lower left panel), or of CVB3-infected mice receiving PBS (CVB3+PBS; upper right panel) or CAPs (CVB3+CAPs; lower right panel), at 200× magnification. MNCs were isolated from the spleen of control mice (open bar graph) or CVB3-infected (closed bar graph) mice. Next, carboxyfluorescein succinimidyl ester-labeled MNCs were directly stimulated with PMA and ionomycin and cultured with or without CAPs (untreated or 24 h pre-treated with L-NAME), in the presence or absence of 1 µg/ml of anti-human IL-10 or anti-murine IFN-γ antibody for 72 h. Then, cells were stained with monoclonal anti-CD4 or anti-CD8 antibodies, followed by flow cytometry and analysis with FlowJo 8.7. software. Bar graphs represent the division index of **C.** CD4+ (upper panel) and of **D.** CD8+ T cells (lower panel) with n = 4/group.

**Figure 7:**
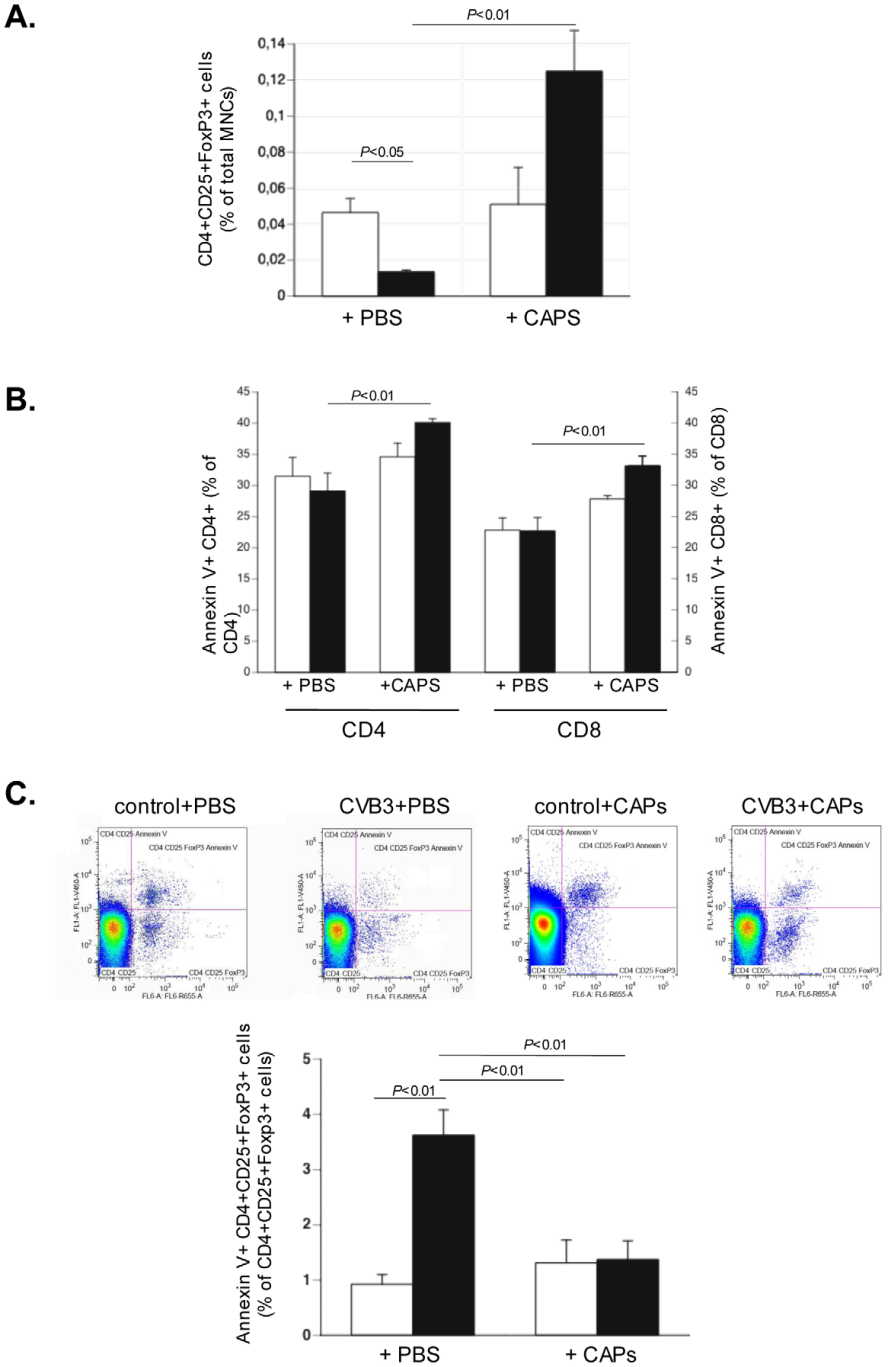
Cardiac adherent proliferating cells increase T regulatory cells, increase apoptotic CD4 and CD8 T cells, and decrease apoptotic T regulatory cells in Coxsackievirus B3-infected mice. Bar graphs represent the mean ± SEM of **A.** T regulatory (CD4CD25FoxP3+) cells depicted as the % of MNCs, in the spleen of control mice (open bars) and CVB3-infected mice (closed bars) injected with PBS or CAPs, as indicated, with n = 4/group. **B.** apoptotic (Annexin V+) CD4+ and CD8 T+ cells, depicted as % of CD4+ or CD8+ T cells, respectively, in the spleen of control mice (open bars) and CVB3-infected mice (closed bars) injected with PBS or CAPs, as indicated, with n = 4/group. **C.** Panel demonstrates representative dot plots of CD4CD25FoxP3+ AnnexinV+ cells from preselected CD4+CD25+cells. Bar graphs representing apoptotic (Annexin V+) T regulatory (CD4CD25FoxP3+) cells depicted as the % of CD4CD25FoxP3+ cells, in the spleen of control mice (open bars) and CVB3-infected mice (closed bars) injected with PBS or CAPs, as indicated, with n = 4/group.

**Figure 8:**
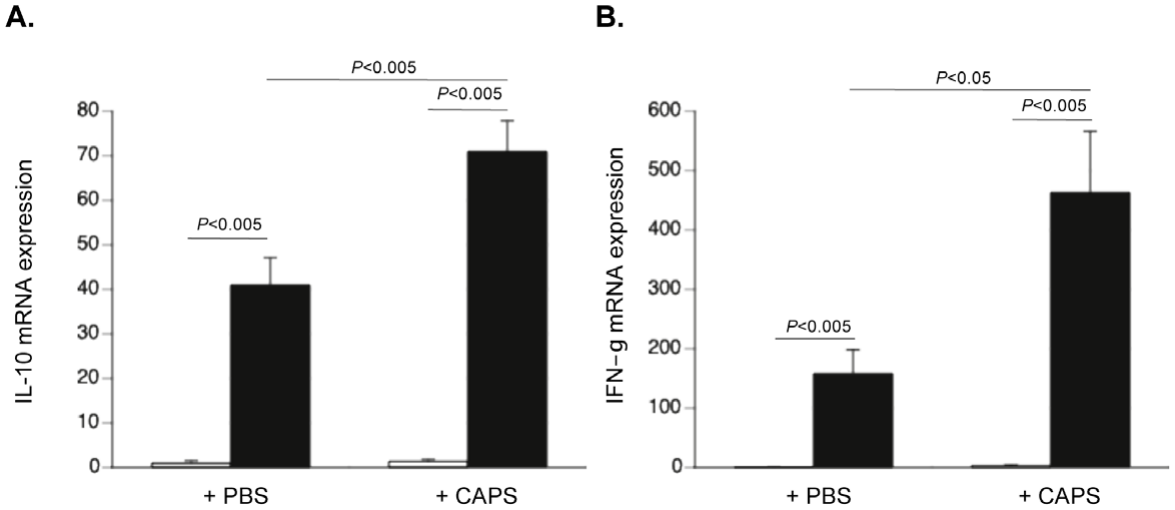
Cardiac adherent proliferating cells increase left ventricular *interleukin-10* and *interferon-γ* mRNA expression in Coxsackievirus B3-infected mice. Bar graphs represent the mean ± SEM of LV **A.** *IL-10* and **B.** *IFN-γ* mRNA expression of control mice (open bars) and CVB3-infected mice (closed bars) injected with PBS or CAPs, as indicated, with n = 8/group.

Given the importance of immune cells in the severity and development of myocarditis [31], we analyzed whether and how CAPs have immunomodulatory effects. *In vivo*, CAPs application reduced the CVB3-induced proliferation/activity of cardiac MNCs in murine acute CVB3-induced myocarditis by 2.9–fold (p<0.05) (**Figure 6A**) and was associated with less cardiac damage (**Figure 6B**). To further investigate underlying mechanisms, MNCs were isolated from the spleen of PBS-injected non-infected control or CVB3-infected mice, carboxyfluorescein succinimidyl ester-labeled and then cultured with or without untreated or L-NAME treated CAPs, or with CAPs in the presence or absence of either 1 µg/ml of anti-murine IFN-γ or anti-human IL-10 antibody. Supplementation of CAPs to MNCs at a ratio of 1 to 10 reduced the PMA/ionomycin-stimulated division index of CD4+ and CD8+ T cells from CVB3-infected mice by 2.7–fold (p<0.01) and 2.3–fold (p<0.001), respectively. This effect was less pronounced or abrogated when CAPs were pre-treated with L-NAME or co-cultured with MNCs in the presence of an anti-IFN-γ or anti-IL-10 antibody, respectively (**Figure 6C and 6D**). Furthermore, CAPs induced the percentage of T regulatory cells (**Figure 7A**), and of apoptotic CD4+ and CD8+ T cells (**Figure 7B**) in the spleen of CVB3-infected mice by 9.3–fold (p<0.05), 1.4–fold (p<0.01) and 1.5–fold (p<0.01), respectively. Importantly, CAPs application decreased the 3.9-fold CVB3-induced (p<0.01 vs. control-PBS) apoptosis of T regulatory cells towards levels not significantly different from PBS-injected control mice (**Figure 7C**). Left ventricle *IL-10* and *IFN-γ* mRNA expression was increased 1.7–fold (p<0.005) and 2.9–fold (p<0.05) in CVB3+CAPs versus CVB3+PBS mice (**Figure 8A and 8B**).

### Interferon-γ and Coxsackievirus B3 increase interleukin-10 but not nitric oxide in cardiac adherent proliferating cells

**Figure 9:**
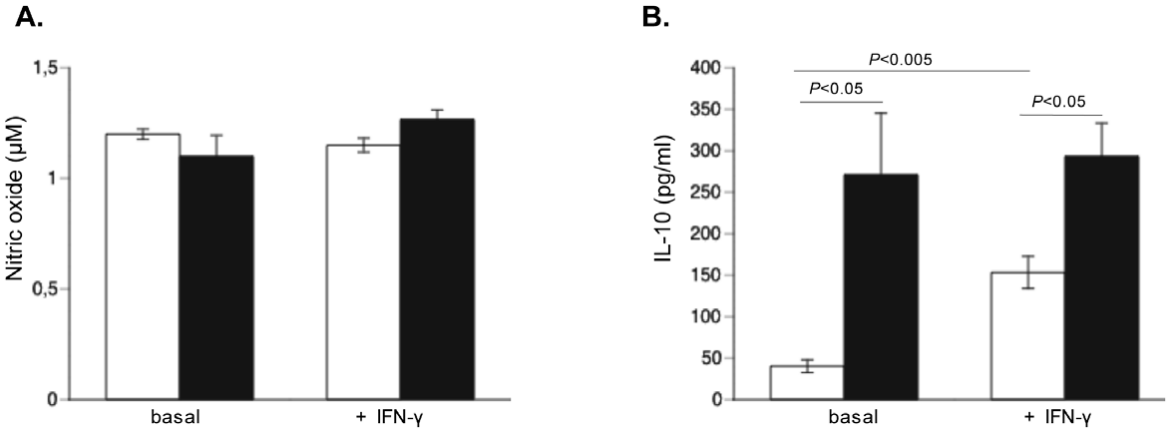
Interferon-γ and Coxsackievirus B3 increase interleukin-10 but not nitric oxide in cardiac adherent proliferating cells. Bar graphs represent the mean ± SEM of **A.** NO (µM) or **B.** IL-10 (pg/ml) production in non-infected or CVB3-infected CAPs with or without supplementation of 4 pg/ml of recombinant murine IFN-γ, with n = 6/condition.

To understand why IFN-γ is important for CAPs to exert their protective effects, we added murine recombinant IFN-γ to uninfected or CVB3-infected CAPs and analyzed the impact of IFN-γ supplementation on NO and IL-10 production. Supplementation of murine IFN-γ to uninfected or CVB3-infected CAPs did not increase NO production. Also CVB3 infection did not influence NO production (**Figure 9A**). In contrast, murine IFN-γ administration raised IL-10 by 3.8-fold (p<0.005) in uninfected CAPs, whereas IL-10 production was 6.7–fold (p<0.01) higher in CVB3-infected versus uninfected CAPs (**Figure 9B**).

### Cardiac-adherent proliferating cells improve left ventricular contractility parameters in experimental Coxsackievirus B3-induced myocarditis

**Figure 10:**
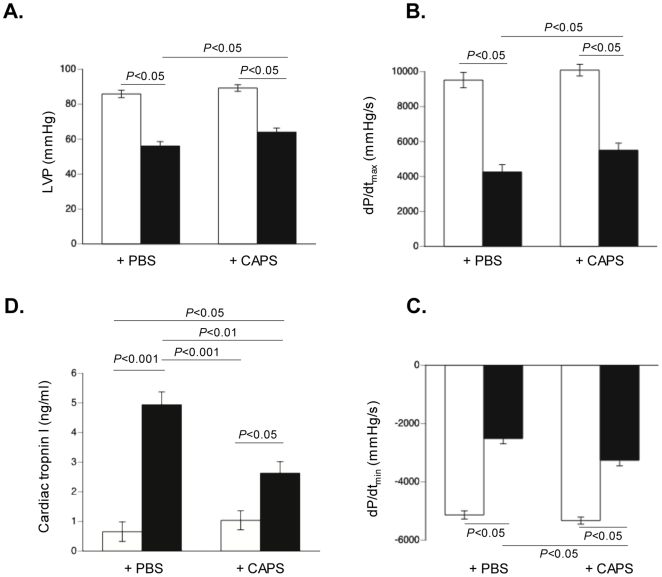
Cardiac adherent proliferating cells improve left ventricular pressure and contractility parameters, and decrease cardiac troponin I levels in murine acute Coxsackievirus B3-induced myocarditis. From A to D: clockwise. Bar graphs representing **A.** left ventricular pressure (mmHg), **B.** dP/dt_max_, and **C.** dP/dt_min_ (mmHg/s) of control mice (open bars) and CVB3-infected mice (closed bars) injected with PBS or CAPs, as indicated (control mice: n = 14; control-CAPs mice: n = 14; CVB3 mice n = 10, and CVB3-CAPs mice: n = 10). **D.** Bar graphs represent cardiac troponin I levels in serum of control mice (open bars) and CVB3-infected mice (closed bars) injected with PBS or CAPs, as indicated, with n = 5/group.

Finally, we evaluated whether the reduction in cardiac apoptosis, viral load and MNC activation after CAPs application in CVB3-infected mice was associated with an improvement in LV contractility. CAPs-treated CVB3-infected mice had significantly improved cardiac contractility and diastolic relaxation compared with CVB3-infected mice receiving only PBS, as indicated by a 1.1–fold (p<0.05) increase in LV pressure and a 1.3–fold (p<0.05) and 1.3–fold (p<0.01) improvement in dP/dt_max_ and dP/dt_min_, respectively (**Figure 10A–C**). In parallel, CVB3-infected mice injected with CAPs had 2.0-fold (p<0.01) lower serum concentrations of the biomarker cardiac troponin I compared to PBS-injected CVB3-infected mice (**Figure 10D**).

### Engraftment of cardiac-adherent proliferating cells after intravenous injection

**Figure 11:**
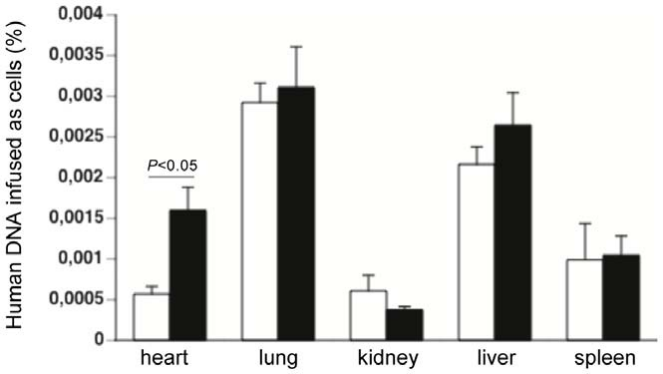
Engraftment of cardiac adherent proliferating cells after intravenous injection. Bar graphs represent human DNA infused as cells (%) in the heart, lung, kidney, liver, and spleen of control mice (open bars) and CVB3-infected mice (closed bars) i.v. injected with CAPs, with n = 4/group.

We evaluated the presence of CAPs in the heart, lung, kidney, liver, and spleen after intravenous injection of CAPs in control and CVB3-infected mice via the detection of human *Alu* sequences (**Figure 11**). CAPs were retrieved in the heart, lung, kidney, liver, and spleen, with highest entrapment found in the lung. Interestingly, there was a 2.8-fold (p<0.05) higher engraftment of CAPs in the heart of CVB3-infected compared to control mice.

## Discussion

The findings of the present study are that CAPs i) cannot be infected by CVB3, ii) reduce CVB3-induced HL-1 cardiomyocyte apoptosis and viral progeny release *in vitro*, in the absence of immune cells, iii) have immunomodulatory features, iv) lead to a decrease in CVB3-induced cardiac apoptosis, viral load and an improvement in LV contractility parameters in murine acute CVB3-induced myocarditis. CAPs exert their protective effects in an NO- and IL-10-dependent manner and require IFN-γ for their activation.

CVB3-induced viral myocarditis is associated with cardiomyocyte apoptosis [1], [4] and impaired LV function [32]. Cardiomyocyte apoptosis can be caused by a direct cytopathic effect of CVB3 [1] or through immune-mediated mechanisms [4]. CAPs have similarities with MSCs [12], [13], which have been demonstrated to have anti-apoptotic [11] and immunomodulatory [14] characteristics, and can reduce CVB3-induced [15] and autoimmune [16] myocarditis. The aim of the present study was to verify whether the newly identified cardiac-derived CAPs share these features and whether they have the potential to be used as a cell source to reduce acute CVB3-induced inflammatory cardiomyopathy.

In order to be considered for clinical use, CAPs or any other cell type should not propagate CVB3 when used for cardiac cell therapy. We demonstrated that CAPs only minimally express CAR [22] and CD55 [23] receptors, which are both critical determinants of cellular uptake and pathogenesis of CVB3 [33]. In accordance with this finding, that suggests minimal CVB3 uptake, CVB3 infection of CAPs was associated with limited CVB3 RNA copy number presence. Furthermore, CVB3 did not reduce the viability of CAPs and viral progeny release did not take place, suggesting that CVB3 propagation would not occur when CAPs are applied in patients with acute CVB3 infection.

In addition, we could demonstrate *in vitro* that CAPs reduce CVB3-induced cardiomyocyte apoptosis and viral progeny release. Since viral progeny release requires apoptosis of infected cells [34], we suggest that the CAPs-mediated reduction in cardiomyocyte apoptosis underlies the decrease in viral progeny release in HL-1 cells and contributes to the decrease in viral load in CVB3-infected mice. Since T cells play an important role in the severity of cardiac damage in CVB3-induced myocarditis [31], we speculate that the observed decrease in cardiac apoptosis and subsequent viral load in CVB3+CAPs mice versus CVB3+PBS mice is due not only to the direct anti-apoptotic effects of CAPs, but also to their immunomodulatory features.

Indeed, application of CAPs in CVB3-infected mice reduced cardiac MNC activation, increased CD4+ and CD8+ T cell apoptosis in the spleen, and raised the % of T regulatory cells, which recently have been shown to protect against CVB3-induced myocarditis [35]. T regulatory cells induce T cell apoptosis and reduce T cell proliferation [36], suggesting that the CAPs-mediated rise in T regulatory cells contributed to the observed decrease in cardiac MNC proliferation/activity and increase in T cell apoptosis. Importantly, CAPs not only increased the amount of T regulatory cells, they also increased their quality, i.e. CAPs reduced the CVB3-induced apoptosis of CD4+CD25+FoxP3+ T cells. CAPs also raised LV *IL-10* and *IFN-γ* mRNA expression. IL-10 reduces cardiomyocyte apoptosis [30] and increases survival in experimental myocarditis [37]. IFN-γ has been shown to reduce CVB3 replication and CVB3-induced cytopathogenicity, in part via the induction of NO release by macrophages [38], [39]. Interestingly, both IL-10 [40] and IFN-γ [36] are crucial in the functionality of T regulatory cells, further supporting the role of induced T regulatory cells in the CAPs-mediated protective effects.

*In vitro*, we showed that CAPs mediated their anti-apoptotic and immunomodulatory effects in an NO- and IL-10-dependent manner. This observation is supported by the evidence that NO and IL-10 exert anti-apoptotic effects on cardiomyocytes [27], [28], [30], play an important role in immunomodulation [14], [41], and improve CVB3-induced myocarditis [29], [37]. This finding corroborates the similarity of CAPs to MSCs, which have previously been shown to exert their anti-apoptotic effects via NO [15] and to suppress T cell proliferation via NO [14], [15] and IL-10 [42].

Furthermore, similar to MSCs [14], [15], CAPs require priming via IFN-γ to exert their anti-apoptotic and immunomodulatory features. However, in contrast to MSCs in which IFN-γ is critical for NO production [43], NO was not induced in CAPs upon IFN-γ supplementation. However, a 3.8-fold rise in IL-10 production by CAPs was observed upon IFN-γ addition. Also CVB3 infection significantly increased IL-10 release, further emphasizing CAPs as an important cell source of IL-10. The prominence of IFN-γ priming for the functionality of CAPs on the one hand, and the increased cardiac IFN-γ levels in acute CVB3-induced myocarditis on the other hand, support the hypothesis that CAPs will exert their protective effects in the cardiac inflammatory environment generated by acute CVB3-induced myocarditis. The increase in cardiac *IFN-γ* mRNA expression upon CAPs application suggests an autonomous self-supporting mechanism.

Our engraftment study could identify CAPs in the heart, lung, liver, kidney, and spleen after intravenous injection. Furthermore, an increased engraftment of CAPs could be found in the hearts of CVB3-infected versus control mice, indicating that CAPs have the capacity to home to the inflamed heart. We believe that the off-target homing of CAPs to the spleen is beneficial. We foresee that the immunomodulatory effects of CAPs in the spleen, e.g. the induction of apoptosis in T cells, which would otherwise invade the myocardium and cause cardiac damage or potentially transfer viral particles into the heart, could contribute to the cardioprotective effects of CAPs in inflammatory cardiomyopathy. This hypothesis is supported by the findings that splenectomy improves the myocardial infarction outcome [44] and that the use of an antibody against T cells reduces the cardiac damage in myocarditis [31]. Furthermore, we suggest that CAPs are able to exert beneficial effects via the release of paracrine factors, such as IL-10 in an analagous manner to MSCs, which are also trapped in the lungs after i.v. injection [45]. Consequently, we suggest that the reduction in cardiac damage and the improvement in LV contractilty after CAPs application in CVB3-induced inflammatory cardiomyopathy is due to the combined protective actions of CAPs in the heart and in the spleen and due to paracrine effects of CAPs located in off-target organs.

In conclusion, our data suggest that CAPs are promising tools to improve acute CVB3-induced inflammatory cardiomyopathy since CAPs i) cannot be infected with CVB3, ii) have anti-apoptotic, anti-viral, and immunomodulatory effects, and iii) improve cardiac contractility parameters in an experimental model of acute CVB3-induced myocarditis.

## Materials and Methods

### Cardiac adherent proliferating cell isolation

Cardiac adherent proliferating cells (CAPs) were isolated from endomyocardial biopsies taken from the right ventricular side of the interventricular septum [24]. CAPs were prepared from biopsies of 3 patients who had chest discomfort and regular ejection fraction. A significant myocardial disorder in those patients had been excluded due to the endomyocardial biopsy analysis. CAPs were harvested as described previously [12]. The donation of cardiac tissue was approved by the ethical committee of the Charité-Universitätsmedizin Berlin (No 225-07) and was obtained with the written consent of the patients.

### Cell culture

Human CAPs were cultured at a density of 6000 cells/cm^2^ in medium consisting of equal amounts of IMDM (PAA)/DMEM/Ham's F12 medium (Biochrom, Berlin, Germany) containing 5% human serum, 1% penicillin/streptomycin, 20 ng/ml basic fibroblast growth factor (Peprotech, Hamburg, Germany) and 10 ng/ml epithelial growth factor (Peprotech). Human cardiac fibroblasts were cultured in Lung/Cardiac Fibroblasts Basal Medium (Cell Applications, Inc. San Diego, USA) plus supplements (CELL Applications). CAR and CD55 expression flow cytometry analysis was performed on CAPs and cardiac fibroblasts of the same passage number. Murine HL-1 cells were cultured in Claycomb medium (SAFC Biosciences, Kansas, USA) supplemented with 10% fetal bovine serum (FBS), 1% penicillin/streptomycin, 100 µM norepinephrine (Sigma, Steinheim, Germany) and 2 mM glutamine. Chinese hamster ovary (CHO) cells and CHO cells expressing human CAR (a kind gift of J.M. Bergelson, Children's Hospital of Philadelphia, Philadelphia) were cultured in Hams F12 medium (PAA, Pasching, Austria) with 10% FBS and 1% penicillin/streptomycin. The CAR antibody-producing hybridoma cell line RMCB (kindly provided by M. Anders, Department of Interdisciplinary Endoscopy, University Hospital Hamburg Eppendorf, Germany) was cultured in suspension in RPMI 1640 medium with 10% FBS and 1% penicillin/streptomycin.

### Surface marker expression

CAPs grown in culture were washed with cold FACS buffer and then labeled with antibodies in 50 µl volume for 25 minutes at 4°C. To exclude non-MSC and dead cells, all cells were labeled with Live/Dead® Fixable Dead Cell Stain violet fluorescent dye (Invitrogen/Molecular Probes), and mouse anti-human monoclonal antibodies CD11b-V450, CD14-V450, CD19-V450, CD34-V450, CD45-V450 (BD Pharmingen). In addition, cells were labeled with CD90-PerCPCy5.5, CD166-PE, CD44-PECy7, CD73-APC, and CD105-FITC (all from Biolegend). Cells were fixed with 200 µl of cold 1% PFA until FACS analysis using a BD FACSCanto II and BD FACSDiva software version 6.1.3. Re-analysis of FACS data was performed using FlowJo software version 8.8.6. (Tree Star Inc.).

### Coxsackievirus and adenovirus receptor and CD55 analysis

CAR and CD55 expression on CAPs, cardiac fibroblasts and HL-1 cells were analyzed by flow cytometry. CHO and CHO-CAR were used as negative and positive controls, respectively. CAR analysis was performed with a mouse anti-CAR antibody generated from *the hybridoma cell line RMCB*, followed by Cy3-labeled anti-mouse secondary antibody staining. As negative control, cells incubated with only the secondary antibody were used. CD55 analysis was conducted with a FITC-labeled mouse anti-human CD55 antibody. As negative control, a FITC-IgG2A isotype control antibody was used.

### Coxsackievirus B3 timeframe experiment with cardiac adherent proliferating cells

CAPs were plated into 6-well plates at a density of 350,000 cells/well. After 24 h of culture, cells were serum starved or infected with CVB3 at an m.o.i. of 5 for 1 h. Then, CAPs were washed with PBS two times and complete CAPs medium was added. Phase contrast pictures at a magnification of 100× were taken 4 h, 12 h, 24 h, and 48 h after serum starvation or CVB3 infection with a Leica camera (Version Twain 7.0.0.0; Leica Microsystems, Wetzlar, Germany) connected with a Leica DMI 4000B microscope (Leica Microsystems, Bensheim, Germany). At the indicated time-points, cells were collected in Trizol for later isolation of RNA. Twenty-four h post CVB3-infection, medium was collected for plaque assay. In parallel, HL-1 cells were infected with CVB3 at an m.o.i. of 5 for 1 h, and cells were collected 4 h, 12 h, and 24 h post-infection in Trizol.

### MTS Viability Assay

10,000 CAPs were plated in each well of a 96-well plate. After 24 h of culture, cells were serum starved or incubated with CVB3 at an m.o.i. of 5 for 1 h. Next, cells were washed 2 times in PBS (Biochrom) and 100 µl of medium was added. 4 h, 12 h, 24 h, and 48 h after serum starvation or CVB3 infection, 20 µl of the CellTiter 96® AQueous One Solution Reagent (Promega, Madison, USA) was added directly to the culture wells, and incubated for 2 h. The absorbance was recorded at 490 nm with a VersaMax microplate reader (Molecular Device GmbH, Munich, Germany).

### Real-time PCR

To analyze CVB3 copy number, and murine *IL-10* and *IFN-γ* mRNA expression, quantitative real-time reverse transcriptase (RT)-PCR (Eppendorf Mastercycler epgradient realplex, Hamburg, Germany) was performed. CVB3 copy number was determined using absolute standard quantification and normalized against murine L32 for HL-1 and human L32 [15] for CAPs. Left ventricular murine *IL-10* and *IFN-γ* mRNA expression was normalized to murine L32 and expressed in relation to the control group (control+PBS mice) set as 1. The following primer pairs were used: CVB3: FOR 5′-CCCTGAATGCGGCTAATCC-3′ and REV 5′-ATTGTCACCATAAGCAGCCA-3′, murine IL-10: FOR 5′-GCCCCAGGCAGAGAAGCATGG-3′ and REV: 5′-GGGAGAAATCGAT GACAGCGCCT-3′, murine IFN-γ: FOR 5′-TCAAGTGGCATAGATGTGGAAGAA-3′ and REV 5′-TGGCTCTGCAGGATTTT CATG-3′, and murine L32 FOR 5′-TGCCCACGGAG GACTGACA-3′ and REV 5′-AGGTGCTGGGAGCTGCTACA-3′.

### Co-culture of HL-1 with cardiac adherent proliferating cells

DiO-labeled HL-1 cells were plated into 6-well plates at a density of 300,000 cells/well for Annexin V/7AAD flow cytometry. After 24 h of culture, HL-1 cells were serum starved or infected with CVB3 at an m.o.i. of 5 for 1 h. Then, cells were washed two times with PBS and complete Claycomb medium was added. Four hours after CVB3-infection or serum starvation, untreated CAPs, or CAPs 24 h pretreated with 10 mM of Nitro-L-Argininmethylesterhydrochloride (L-NAME; Sigma) were collected and added to the HL-1 cells for co-culture at a ratio of CAPs to HL-1 of 1 to 10, which is a commonly used ratio of MSCs versus target cells [14], [17]. To investigate whether CAPs mediate their effects via interleukin (IL)-10, 1 µg/ml of anti-human IL-10 antibody or a corresponding isotype control (R&D Systems, Minneapolis, USA) was added at the timepoint of CAPs supplementation. To investigate the role of IL-10 secreted by CAPs in the CAPs-mediated protective effects, 6 pg/ml of recombinant human IL-10 (R&D Systems), corresponding to the concentration of IL-10 secreted by CAPs in HL-1-CAPs co-cultures, was supplemented to uninfected and CVB3-infected HL-1 cells. To evaluate whether the CAPs-mediated effects require IFN-γ, anti-mouse IFN-γ antibody or its isotype control (R&D Systems) was added at a final concentration of 1 µg/ml. At these concentrations, respective isotype controls did not affect the CAPs-mediated effects (data not shown), neither did anti-human IL-10 antibody or anti-mouse IFN-γ antibody affect apoptosis in uninfected or CVB3-infected HL-1 cells in the absence of CAPs (data not shown). Twenty-four hours after infection, phase contrast pictures were taken, supernatant was collected for plaque assay, and cells were collected for analysis by flow cytometry.

### Annexin V/7AAD analysis

To be able to detect apoptosis specifically in HL-1 cells in the HL-1/CAPs co-cultures, HL-1 cells were labeled with Vybrant® DiO Cell-labeling (Invitrogen, Heidelberg, Germany) before plating. Twenty-four hours after CVB3 infection, cells were collected, fixated in paraformaldehyde and resuspended in PBS for flow cytometry analysis. Apoptosis was analyzed with the Annexin V-PE Apoptosis Detection Kit (BDSciences, Franklin Lakes, USA) with Annexin V-positive and 7-Amino-Actinomycin (7-AAD) negative cells considered apoptotic. Annexin V/7AAD on DiO+ gated cells was analyzed by fluorescence-activated cell sorting (FACS) using a FACSScan flow cytometer and Cell Quest software (BD Biosciences, San Jose, CA, USA). Data are expressed as DiO+/Annexin V+/7AAD- cells (% gated).

### Coxsackievirus B3 plaque assay

HeLa cells (DSMZ, Braunschweig, Germany) were plated in 6-well plates (350,000/well) and cultured in RPMI 1640 (Invitrogen), supplemented with 10% FBS and 1% penicillin/streptomycin. Serial dilutions of supernatant from CVB3-infected CAPs and from (co)-cultures of CVB3-infected HL-1 cells with or without CAPs in the presence or absence of anti-human IL-10 or anti-murine-IFN-γ antibodies or with CAPs pretreated with L-NAME, and of LV tissue homogenates from CVB3-infected mice and CVB3-infected mice injected with CAPs, were prepared. After culture of HeLa cells for 24 h, the medium was removed and cells were washed with PBS. Next, 1 ml of the prepared dilutions was added to each well and incubated for 30 min at 37°C. Then, 2 ml agar, consisting of an equal volume of MEM2x, supplemented with 4% FBS, and of 1.3% noble agar, was added to each well. The plates were left under the hood for 15–20 min until the agar coagulated, and then kept in the incubator for 72 h. Finally, virus plaques were counted in n = 6 wells/*in vitro* condition or as indicated, and in 12 wells/*in vivo* group (3 wells/mouse and n = 4 mice/group).

### Nitric oxide analysis

10,000 CAPs were plated in each well of a 96-well plate. After 24 h of culture, cells were serum starved or incubated with CVB3 at an m.o.i. of 5 for 1 h. Next, cells were washed 2 times in PBS (Biochrom) and 100 µl of medium was added. Four hours later, recombinant murine IFN-γ (R&D Systems) was added at a final concentration of 4 pg/ml, corresponding to the concentration of murine IFN-γ present in medium of HL-1 cells and HL-1/CAPs co-cultures (data not shown). Twenty-four hours after serum starvation or CVB3 infection, medium was collected for NO analysis. NO was determined with a commercial NO kit according to the manufacturer's protocol (Calbiochem, Darmstadt, Germany). Fluorescence was measured with a fluorometer (Berthold Technologies, LB 940 Multimode Reader Mithras, Bad Wildbad, Germany) at excitation and emission wavelengths of 365 nm and 450 nm, respectively.

### Interleukin-10 ELISA

CAPs were plated into 6-well plates at a density of 350,000 cells/well. After 24 h of culture, CAPs were serum starved or infected with CVB3 at an m.o.i. of 5 for 1 h. Then, cells were washed two times with PBS and complete CAPs medium was added. Four hours later, recombinant murine IFN-γ (R&D Systems) was added at a final concentration of 4 pg/ml, corresponding to the concentration of murine IFN-γ present in medium of HL-1 cells and HL-1/CAPs co-cultures (data not shown). Twenty-four hours after serum starvation or CVB3 infection, medium was collected for IL-10 analysis. Human IL-10 was analyzed with an IL-10 ELISA kit (R&D Systems) according to the manufacturers protocol.

### Animals

The low immunogenicity of CAPs [21] and our finding that co-culture of human CAPs with murine splenocytes did not result in splenocyte proliferation/activation (data not shown) prompted us to evaluate the effect of CAPs administration in a murine model of acute myocarditis. To study the effect of CAPs application on the progression of CVB3-induced myocarditis, 10^6^ CAPs or PBS was i.v. [46] injected in 6- to 8-week-old C57BL/6 mice, one day after i.p. infection with 5×10^5^ plaque-forming units (p.f.u.) of CVB3 (Nancy strain) (CVB3-CAPs versus CVB3 mice, respectively). Uninfected controls received PBS instead of CVB3. Seven days after CVB3 infection, hemodynamic parameters were analyzed, followed by harvesting of the LV, which was next snap-frozen for performing molecular biology, viral load analysis and immunohistochemistry. For the analysis of MNC proliferation, the heart and spleen were isolated. To evaluate the engraftment of CAPs after i.v. injection, the heart, spleen, lung, kidney, and liver were isolated. The investigation was performed in accordance with the principles of laboratory animal care and the German law on animal protection and with the approval of the ethical committee for the use of experimental animals of the Charité-Universitätsmedizin Berlin (No G0094/01).

### Hemodynamic measurements

Seven days after CVB3 infection, mice were anesthetized (0.8–1.2 g/kg urethane and 0.05 mg/kg buprenorphine intraperitoneally) and artificially ventilated. A 1.2F microconductance pressure-volume catheter (Scisense Inc., Ontario, Canada) was positioned in the LV through the apex for continuous registration of real-time pressure-volume loops in an open chest model [15]. The volumes were calibrated using the hypertonic (10%) saline wash-in technique. All measurements were performed three times during which ventilation was momentarily turned off. Systolic function and myocardial contractility were quantified by LV end-systolic pressure and peak rate of rise of LV pressure (dP/dt_max_). Diastolic performance was measured by peak rate of LV pressure decrease dP/dt_min_.

### Histology and TUNEL Staining

Hematoxylin and eosin staining was performed on 5 µm thick cryosections. Apoptotic cells were detected on 5 µm thick cryosections by end labeling the fragmented DNA using the DeadEnd Colorimetic TUNEL System (Promega) according to the manufacturer's instructions.

### Caspase 3/7 Assay

LV caspase 3/7 activity was measured with a caspase-Glo 3/7 assay kit (Promega) according to the manufacturer's protocol. In brief, 100 µL containing 30 µg of LV protein extracts of C57BL/6 control and CVB3 infected mice i.v. injected with CAPs or PBS were added to a white-walled 96-well luminometer plate. Then, 100 µL of caspase-Glo 3/7 reagent containing caspase 3/7 buffer and the proluminescent caspase 3/7 substrate, was added to each sample. After 30 min of incubation at room temperature, the luminescence of each sample was measured in a microplate-reading luminometer (Mithras LB 940, Berthold Technologies GmbH & Co KG, Germany). Data represent the absorbance of the samples minus the absorbance of the protein extraction buffer as control.

### (Cardiac) mononuclear cell, CD4+, and CD8+ T cell proliferation

Cardiac MNCs were isolated from control+PBS, control+CAPs, CVB3+PBS and CVB3+CAPs mice, 7 days post-infection via trypsinization of heart pieces over night, followed by enzymatic digestion with collagenase type 2 (0.25 mg/ml; Sigma, Steinheim, Germany), sequential centrifugation at low speed to sediment the large cells/cardiomyocytes, and further separation by density gradient sedimentation using Histopaque (Sigma) [15]. A minimum of n = 10 hearts/group were pooled to perform the experiment. Cardiac MNCs were labeled with 10 µM of succinimidyl ester of carboxyfluorescein diacetate (CFSE Cell Tract™; Invitrogen, Carlsbad, CA, USA) to be able to measure cell proliferation, which would indicate MNC activation. Phorbol myristate acetate (PMA; final concentration of 50 ng/ml) and ionomycin (final concentration of 500 ng/ml) were used to stimulate cardiac MNCs *in vitro*. Cardiac MNCs were cultured in RPMI1640 medium (Invitrogen, Heidelberg, Germany), supplemented with 10% FBS and 1% penicillin/streptomycin for 72 h, followed by flow cytometry using a MACSQuant Analyzer (Miltenyi Biotec, Bergisch Gladbach, Germany) and analysis with FlowJo 8.7. software (Tree Star). To investigate how CAPs reduce MNC (CD4+ and CD8+ T cell) proliferation, splenocytes were isolated from control and CVB3-infected C57BL/6 mice according to De Geest *et al.* [47]. Next, MNCs were carboxyfluorescein succinimidyl ester-labeled, stimulated with PMA/ionomycin and directly (co)-cultured, with or without CAPs (untreated or 24 h pre-treated with L-NAME) at a ratio of 10∶1 in RPMI1640, 10% FBS, 1% penicillin/streptomycin, in the presence or absence of 1 µg/ml of anti-human IL-10, anti-murine IFN-γ antibody, or respective isotype controls for 72 h. At these concentrations, the isotype controls did not affect the CAPs-mediated effects (data not shown). Supplementation of 1 µg/ml of anti-human IL-10 or anti-murine IFN-γ antibody to stimulated splenocytes alone did not affect their proliferation (data not shown). Then, cells were stained with monoclonal anti-CD4 or anti-CD8 antibodies (BD Biosciences, Franklin Lakes, NJ, USA), followed by flow cytometry on a MACSQuant Analyzer (Miltenyi Biotec, Bergisch Gladbach, Germany) and analysis with FlowJo 8.7. software (Tree Star). For all proliferation analysis mentioned previously, the average number of cell divisions that the responding cells undergo, while excluding the cells of peak 0 that did not divide, was calculated by the software as the division index.

### Analysis of T regulatory cells, apoptotic T regulatory cells, and apoptotic CD4+ and CD8+ T cells

Prior to staining, splenocytes were treated with mouse FcR Blocking Reagent (Miltenyi Biotec), used to block unwanted binding of antibodies to mouse cells expressing Fc receptors. Antibodies to CD4, CD8, CD25, and FoxP3, and the Tregs Detection Kit were purchased from Miltenyi Biotec, and Annexin V-APC and Annexin V-V450 were from BDSciences. For CD4CD25FoxP3 triple immunofluorescence, the cells were stained with CD4-FITC and CD25-PE, and then, after fixation and permeabilization, with FoxP3-APC monoclonal antibodies. Apoptosis in T regulatory cells was detected by Annexin V-V450 co-staining. Therefore, the cells were first stained with CD4-FITC, CD25-PE, and Annexin V-V450 and then, after fixation and permeabilization, with FoxP3-APC monoclonal antibodies. Apoptosis in CD4+ and CD8+ T cells was detected by Annexin V-APC co-staining. All the samples were analysed by flow cytometry analysis (MACSQuant Analyzer, MACSQuantify™ Software, Miltenyi Biotec, Bergisch Gladbach, Germany).

### Analysis of cardiac troponin-I

Mouse cardiac troponin-I levels were determined in murine serum (n = 5/group) with a mouse cardiac troponin-I ELISA kit (Life Diagnostics, West Chester, PA, USA), according to the manufacturers protocol.

### Engraftment assayed by real-time PCR

Levels of human CAPs in the myocardium were quantified according to the method of McBride *et al.* [48] with slight modifications. In brief, genomic DNA was extracted from frozen tissues as described previously [49]. A standard curve was generated using human genomic DNA obtained from HUVECs serially diluted over a 100,000-fold dilution range, into murine spleen genomic DNA. Real-time PCR was performed with 800 ng of target DNA, Alu specific primers and a fluorescent probe [48]. Values are expressed as % of human DNA per 800 ng of murine tissue.

### Statistical analysis

Statistical analysis was performed using GraphPad Instat 3.0a (GraphPad Software, Inc., La Jolla, USA). Assumption of Gaussian distribution was consistently tested by the method of Kolmogorov and Smirnov. Paired and unpaired Student's t tests were used for statistical analysis. When Gaussian distribution was not reached, a non-parametrical test was used. Data are presented as mean ± SEM. Differences were considered to be significant when the two-sided p-value was lower than 0.05.

## Supporting Information

Figure S1 **Flow cytometry analysis of cardiac adherent proliferating cells.** Representative flow cytometry histograms as overlay of the control stained cells (solid black line) and the specifically stained cells with appropriate antibody (filled grey graph) indicate that CAPs are CD11b^−^, CD14^−^, CD19^−^, CD34^−^, CD45^−^, CD90^−^, and CD166^+^, CD44^+^, CD73^+^, and CD105^+^.
(TIF)
