# Supplementary figures and images for: Human Cardiac-Derived Adherent Proliferating Cells Reduce Murine Acute Coxsackievirus B3-Induced Myocarditis
Source: PLoS One. 2011 Dec 9;6(12):e28513. doi: 10.1371/journal.pone.0028513 (PMC3235117; doi:10.1371/journal.pone.0028513)

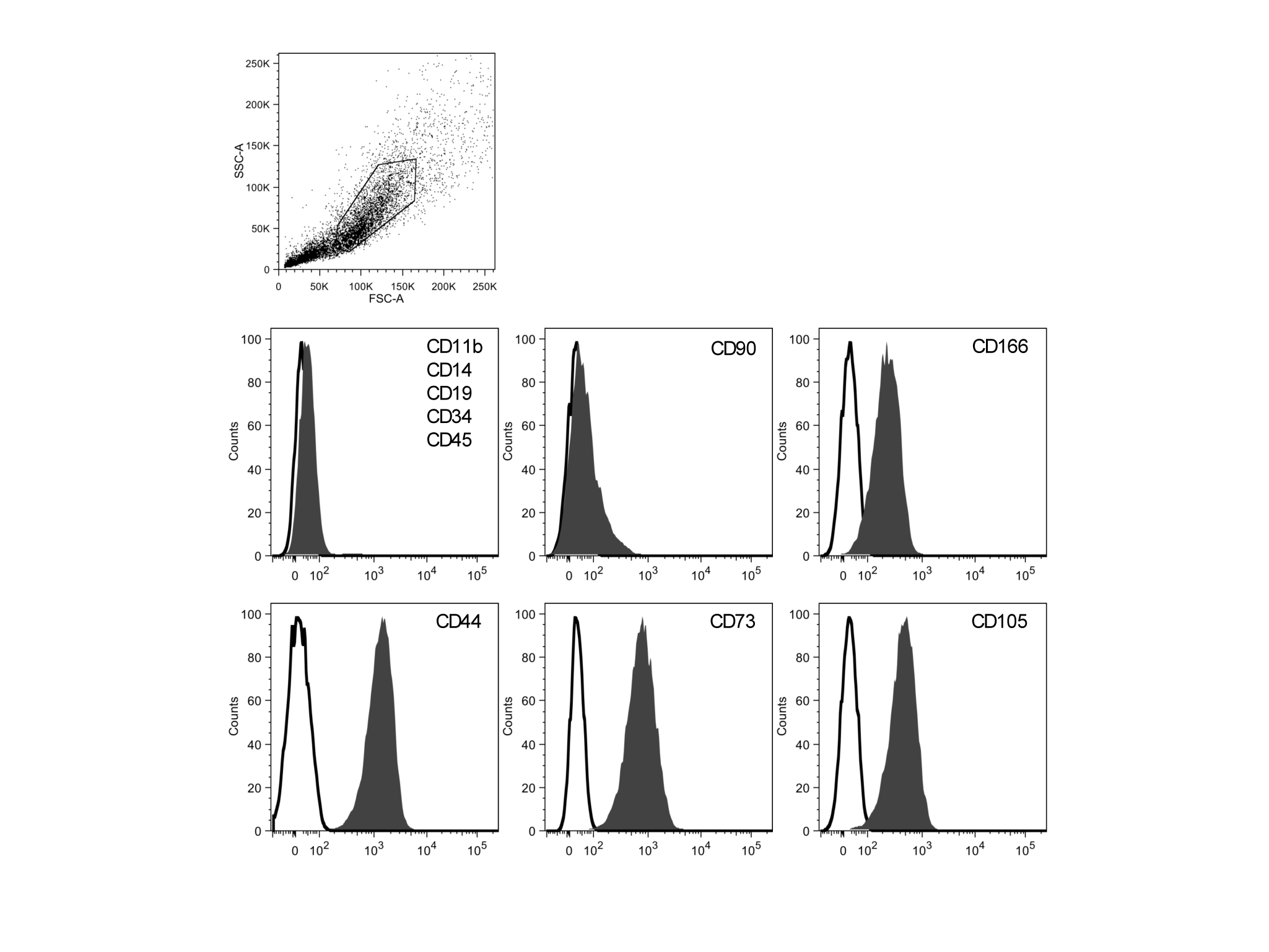

Supplement: Figure S1 — Flow cytometry analysis of cardiac adherent proliferating cells. Representative flow cytometry histograms as overlay of the control stained cells (solid black line) and the specifically stained cells with appropriate antibody (filled grey graph) indicate that CAPs are CD11b−, CD14−, CD19−, CD34−, CD45−, CD90−, and CD166+, CD44+, CD73+, and CD105+. (TIF) [file pone.0028513.s001.tif]
